# Impact of diarrhoea and acute respiratory infection on environmental enteric dysfunction and growth of malnourished children in Pakistan: a longitudinal cohort study

**DOI:** 10.1016/j.lansea.2023.100212

**Published:** 2023-06-07

**Authors:** Azza Sarfraz, Zehra Jamil, Sheraz Ahmed, Fayaz Umrani, Abdul Khaliq Qureshi, Sadaf Jakhro, Muhammad Sajid, Najeeb Rahman, Arjumand Rizvi, Jennie Z. Ma, Indika Mallawaarachchi, Najeeha T. Iqbal, Sana Syed, Junaid Iqbal, Kamran Sadiq, Sean R. Moore, Syed Asad Ali

**Affiliations:** a Department of Pediatrics and Child Health, The Aga Khan University, Pakistan; b Department of Biological & Biomedical Sciences, The Aga Khan University, Pakistan; c Department of Public Health Sciences, University of Virginia, Charlottesville, VA, USA; d Department of Pediatrics, University of Virginia, Charlottesville, VA, USA

**Keywords:** Acute respiratory illness, Diarrhoea, Morbidity, Linear growth, Ponderal growth, Environmental enteric disease

## Abstract

**Background:**

Diarrhoea and acute respiratory infections (ARI) are assumed to be major drivers of growth and likely contribute to environmental enteric dysfunction (EED), which is a precursor to childhood malnutrition. In the present study, we checked the correlation between diarrhoeal/ARI burden and EED using a novel duodenal histological index.

**Methods:**

Between November 2017 and July 2019, a total of 365 infants with weight-for-height Z scores (WHZ score) of <−2 were enrolled, and 51 infants with WHZ scores of >0 and height-for-age Z scores (HAZ scores) of >−1 were selected as age-matched healthy controls. Morbidity was assessed weekly and categorised as the total number of days with diarrhoea and acute respiratory infection (ARI) from enrolment until two years of age and was further divided into four quartiles in ascending order.

**Findings:**

The HAZ declined until two years of age regardless of morbidity burden, and WHZ and weight-for-age Z scores (WAZ scores) were at their lowest at six months. Sixty-three subjects who had a WHZ score <−2 and failed to respond to nutritional and educational interventions were further selected at 15 months to investigate their EED histological scores with endoscopy further. EED histological scores of the subjects were higher with increasing diarrhoeal frequency yet remained statistically insignificant (p = 0.810).

**Interpretation:**

There was not a clear correlation between diarrhoea and ARI frequency with growth faltering, however, children with the highest frequency of diarrhoea had the highest EED histological scores and growth faltering.

**Funding:**

10.13039/100000865Bill and Melinda Gates Foundation and The National Institutes of Health.

**Research in context**

**Evidence before this study**

When this study was initiated in 2016, evidence of environmental enteric dysfunction (EED) in children and its impact on stunting was scarce. While the study was being conducted, we identified articles that examined the link between EED and linear growth faltering in resource-constrained settings. Mechanisms linking EED and stunting were limited with a plausible explanation associated with systemic inflammation and malabsorption. Diarrhoeal disease was one of the suspected environmental/nutritional factors that may cause enteropathy in low- and middle-income countries (LMICs). However, there was no direct evidence linking morbidity frequencies, growth patterns, and EED to the best of our knowledge thus far.

**Added value of this study**

This trial enrolled and followed Pakistani children from birth to 2 years of age to observe the effect of morbidity frequency on growth trends and EED-associated gut histological scores. During the longitudinal trial, the children were assessed for eligibility for education followed by nutritional interventions. Educational interventions were given until 9 months of the child's age. If the child's weight-for-height Z-score remained <−2 SD, nutritional rehabilitation was given with ready-to-use supplementary food (RUSF). Duodenal histology scores were compiled using the semi-quantitative criteria (aggregate score of 37) comprising 11 parameters developed by gastrointestinal pathologists with input from members of the EED Biopsy Initiative Consortium. Our trial findings will generate evidence for guiding future studies to reduce stunting in children two years of age.

**Implications of all the available evidence**

The findings of the trial revealed that the diarrhoeal and acute respiratory infection (ARI) morbidities with the relatively highest frequency had consistently worsened linear and ponderal growth faltering. Our findings also highlighted the correlation of diarrhoeal and ARI morbidity frequency with duodenal histology scores. Diarrhoeal morbidity was linearly related to duodenal histology scores indicative of EED severity. This trial showed that stunting prevention through nutritional intervention with RUSF did not improve growth patterns. We also found that morbidity alone is not enough to explain growth faltering trends. Our findings lend credit to intestinal damage due to underlying EED as a contributor to stunting in children under 2 years in a resource-constrained setting.

## Introduction

Previous studies have shown linear and ponderal growth retardation in children who suffered from diarrhoeal and acute respiratory infection (ARI) morbidities.^1^, ^2^, ^3^, ^4^, ^5^, ^6^, ^7^, ^8^, ^9^ However, the data linking the frequency of diarrhoea and ARI with linear and ponderal growth faltering in children is equivocal. Environmental enteric dysfunction (EED) has been historically linked with malnutrition, growth failure, and poor linear growth particularly in children from low-income and middle-income countries (LMIC).^1^^,^^2^ EED typically affects the gut with underlying pathological events ranging from intestinal inflammation to damage to the intestinal morphology, causing shortened and blunted villi and crypt hyperplasia.^10^ However, no studies have found direct evidence linking the frequency of diarrhoea and ARI to growth faltering and EED-related changes in gut histology. We conducted a longitudinal study to determine the impact of diarrhoea and ARI frequency on growth trends and duodenal histological scores as an indicator of EED severity in children until the age of two years. Our primary hypotheses were that higher diarrhoeal and ARI prevalence correlates with (1) lower weight-for-age z-score (WAZ), height-for-age z-score (HAZ), and weight-for-height z-score (WHZ), and (2) higher aggregate EED histology scores.^11^

## Methods

### Study design

The Study of Environmental Enteropathy and Malnutrition (SEEM) was a longitudinal study with an established field site at Matiari, Pakistan (approximately 190 km from Karachi). The study protocols for SEEM was approved by the Ethical Review Committee (ERC) of Aga Khan University in 2015 (Protocol 3836-Ped-ERC-15). Children were enrolled in the study after obtaining written informed consent from parents or legal guardians. All human subject research ethics were followed in accordance with relevant guidelines and regulations during the entire duration of the studies.

Data for these analyses came from an established birth cohort of 416 children followed for two years between November 2017 and July 2019, as described previously.^12^ The anthropometry data of children were collected monthly. Three hundred sixty-five children with WHZ < −2 and fifty-one age-matched controls with linear anthropometric assessments of WHZ >0 and HAZ > −1 on two consecutive visits between 3 and 6 months were recruited. Parents/guardians received appropriate educational counselling upon enrolment at age <6 months, focusing on breastfeeding best practices. The second educational nutritional interventional phase was initiated during age 6 months-2 years, promoting complementary feeding as best practice. For those children, if their WHZ remained < −2 at nine months, the nutritional rehabilitation phase included AchaMum, a local ready-to-use supplementary food (RUSF). Children were administered the high-calorie AchaMum according to Pakistan's Community Management of Acute Malnutrition protocol based on the WHZ indicator being < −2 at 9–10 months of age until 12 months.

The decision to perform an upper gastrointestinal biopsy and endoscopy was based on an a-priori-determined protocol. Children who remained moderately or severely malnourished (WHZ < −2 or −3 respectively) despite these interventions were evaluated for further clinical workup of malnutrition. The standardised laboratory panel included celiac screening, complete blood count, complete metabolic panel, international normalized ratio, erythrocyte sedimentation rate, and C-reactive protein. Furthermore, the paediatric gastroenterologist ordered any additional tests as clinically indicated. If no cause was apparent on basic laboratory workup, more thorough investigation with gastrointestinal biopsy/endoscopy was conducted at the Aga Khan University Hospital (AKUH). Nutritional intervention non-responders in whom no apparent cause of malnutrition was identified after basic laboratory workup were medically evaluated with an upper gastrointestinal endoscopy and scored for histopathological changes per the geographic-specific index established for our region (the subcontinent),^11^ as deemed necessary by the paediatric gastroenterologist at the AKUH.

### Data collection

Community health workers (CHWs) were trained at the Matiari Research and Training Centre. After enrolment, CHWs visited the homes of the participants every week, interviewed mothers, and filled out a standard form translated into the local language Sindhi. This form contained information about symptoms of diarrhoea and acute respiratory illness. Episodes of diarrhoea or ARI episodes were established when a child demonstrated signs or symptoms for a minimum of 2 days, followed by a 7-day symptom-free interval. A "diarrhoea day" was defined as the passage of three or more loose or liquid stools and an "ARI day" was defined as the maternal perception of cough or shortness of breath. Monthly anthropometric measurements were collected, including weight, length, mid-upper arm circumference (MUAC), and occipitofrontal head circumference.

The duodenal biopsy specimen was obtained for detailed histopathological assessment, and all specimens were stored under standard protocols and stained with haematoxylin and eosin (H&E). Pathologists graded the duodenal biopsies to determine the severity of EED. The pathologists considered all sections from the samples and reported a subjective average as per the EED scoring index histology criteria. The score is a semi-quantitative index comprising 11 parameters commonly implicated in tissue injury and response patterns: acute and chronic inflammation, eosinophilic infiltration, intraepithelial lymphocytes, villous architecture, intramucosal Brunner glands, foveolar cell metaplasia, goblet and paneth cell densities, enterocyte injury, and epithelial detachment. Each of these features had its sub-scores adding up to a total aggregate score of 37. The scores were developed by the EED Biopsy Initiative (EEDBI) Consortium.^11^ The detailed protocol of SEEM has been published previously.^12^

### Data analysis

The primary outcomes were the growth indices calculated by comparing child anthropometric measurements from WHO Child Growth Standards (WHO Anthro, Geneva, Switzerland); specific models include length (SECA 417), weight (SECA 354), mid-upper arm circumference (SECA 212), and head circumference (SECA 212) The scores were assessed as continuous measures of HAZ, WHZ, and WAZ.^13^ The secondary outcome was the histology score. The exposure was the duration of diarrhoea and ARI. The total number of diarrhoea or ARI days from enrolment till two years of age was divided into four quartiles. Data were summarised using frequencies and percentages for categorical variables and mean with standard deviation or median with interquartile range (Q1, Q3) for continuous variables. Diarrhoea and ARI groups were compared using ANOVA for continuous outcomes and a chi-square test for categorical outcomes at each time point. *P*-values were adjusted for multiple testing using Bonferroni correction. All statistical analyses were performed using Stata 17, and *p*-values <0.05 were considered statistically significant.

### Role of the funding source

The funders had no role in the design, data collection, analysis of the study, nor the decision to publish or prepare this manuscript.

## Results

**Fig. 1:**
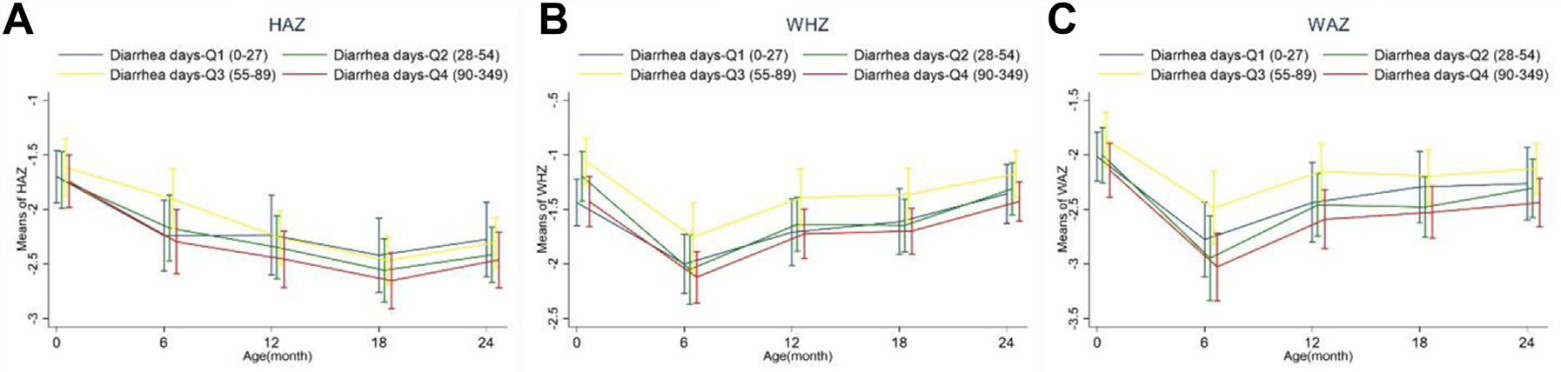
Growth trajectory including A) Height-for-age z-score (HAZ), B) Weight-for-height z-score (WHZ) and C) Weight-for-age z-score (WAZ) of children with different burdens of diarrhoea.

**Fig. 2:**
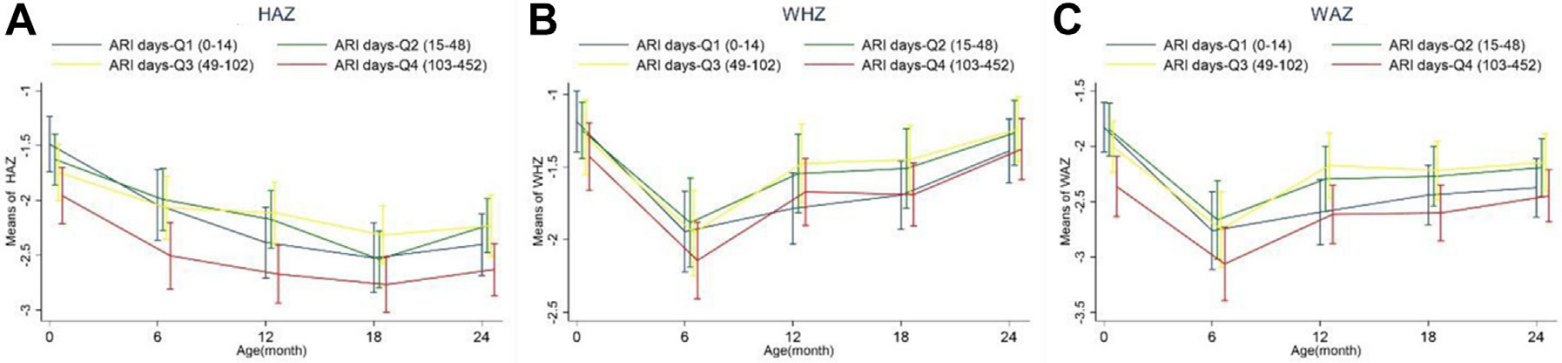
Growth trajectory including A) Height-for-age z-score (HAZ), B) Weight-for-height z-score (WHZ) and C) Weight-for-age z-score (WAZ) of children with different burdens of acute respiratory infection (ARI).

The descriptive statistics of the study population are summarised in Table 1. Across the cohort, 401 participants had morbidity episodes, of which 60% were males with comparable diarrhoea and ARI prevalence across both genders. The overall mean diarrhoea prevalence through 2 years of age among children was 49 days, with the median diarrhoeal episode of 9.46 [6.16, 13.69]. The mean ARI prevalence was 52.4 days throughout enrolment, and the median ARI episodes were 2.92 [1.3, 5.1]. The HAZ score across 24 months of age progressively decreased regardless of diarrhoea or ARI prevalence, with some improvement in the 18–24 months segment (Fig. 1, Fig. 2). WAZ and WHZ scores reached their lowest at six months and gradually improved (Fig. 1, Fig. 2).

**Table 1:** Descriptive statistics of acute respiratory infection (ARI) and diarrhoea prevalence and episodes.

	Male	Female	Overall
**Participants (n)**	241	160	401
**Mean days with diarrhoea per year**^a^	47.9	50.6	49.0
**Mean diarrhoea episodes per year (SD)**^b^	10.0 (5.9)	10.8 (5.9)	10.3 (5.9)
**Median diarrhoea episodes per year [Q1, Q3]**^b^	9.5 [5.8, 13.0]	9.5 [6.7, 15.5]	9.5 [6.2, 13.7]
**Diarrhoea days (Quartiles)**			
**Q1 (0–27)**	67 (27.8%)	37 (23.1%)	104 (25.9%)
**Q2 (28–54)**	56 (23.2%)	41 (25.6%)	97 (24.2%)
**Q3 (55–89)**	61 (25.3%)	39 (24.4%)	100 (24.9%)
**Q4 (90–349)**	57 (23.7%)	43 (26.9%)	100 (24.9%)
**Mean days with ARI per year**^a^	54.9	48.5	52.4
**Mean ARI episodes per year (SD)**^b^	3.5 (2.7)	3.4 (3.5)	3.5 (3.0)
**Median ARI episodes per year [Q1, Q3]**^b^	3.0 [1.5, 5.2]	2.5 [1.2, 4.9]	2.92 [1.3, 5.1]
**ARI days Quartiles**			
**Q1 (0–14)**	56 (23.2%)	48 (30.0%)	104 (25.9%)
**Q2 (15–48)**	65 (27.0%)	37 (23.1%)	102 (25.4%)
**Q3 (49–102)**	57 (23.7%)	40 (25.0%)	97 (24.2%)
**Q4 (103–452)**	63 (26.1%)	35 (21.9%)	98 (24.4%)

a (Diarrhoea days/Observed days) ∗365.

b (Diarrhoea episodes/Observed days) ∗365. ∗15 children did not have any morbidity episode.

We explored the diarrhoeal days among children and their association with growth (Fig. 1). In Q1, children had up to 27 days of diarrhoea from enrolment to 24 months. In Q2, Q3, and Q4, diarrhoeal days were between 28 and 54 days, 55–89 days, and 90–349 days, respectively (Table 1). Children in diarrhoeal Q4 consistently had lower yet statistically insignificant WAZ (p = 0.120, p = 0.170, p = 0.240, p = 0.360), HAZ (p = 0.260, 0.700, 0.660, 0.730), and WHZ (p = 0.290, 0.230, 0.190, 0.410) scores compared to diarrhoeal Q1-Q3 at age 6, 9, 12, 18, and 24 months, respectively (Fig. 1 and Supplementary Table S1); Children in diarrhoeal Q1-2 had worse WAZ, HAZ, and WHZ scores than diarrhoeal Q3 despite insignificant findings (p-values summarized above) (Fig. 1 and Supplementary Table S1).

We similarly explored the ARI days among the children and their association with growth (Fig. 2). In Q1, children had up to 14 days of respiratory symptoms up to 24 months. In Q2, the burden of ARI was 15–48 days. The ARI days across Q3 and Q4 were 49–102 and 102 to 452, respectively (Table 1). Children in ARI Q4 had consistently worse yet statistically insignificant WAZ (p = 0.410, 0.082, 0.150, 0.280), HAZ (p = 0.062, 0.020, 0.110, 0.091), and WHZ (p = 0.620, 0.390, 0.380, 0.720) scores at age 6, 9, 12, 18, and 24 months, respectively (Fig. 2); the HAZ scores at 12 months were significantly lowest in Q4 (p = 0.02). Children in ARI Q1-2 had lower WAZ and WHZ scores than Q3 at age 6, 9, 12, 18, and 24 months despite insignificant results (p-values summarized above) (Fig. 2, Supplementary Table S1). The HAZ scores were worse across children in ARI Q1-2 compared to Q3 at 12 (p = 0.020), 18 (p = 0.110), and 24 (p = 0.091) months (Fig. 2, Supplementary Table S1).

**Fig. 3:**
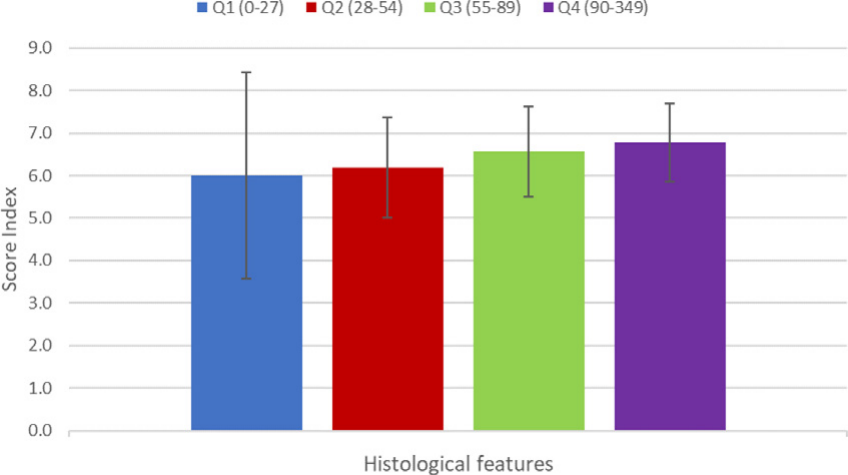
Average biopsy scores in children with different burdens of diarrhoea. Q indicate quartiles.

**Fig. 4:**
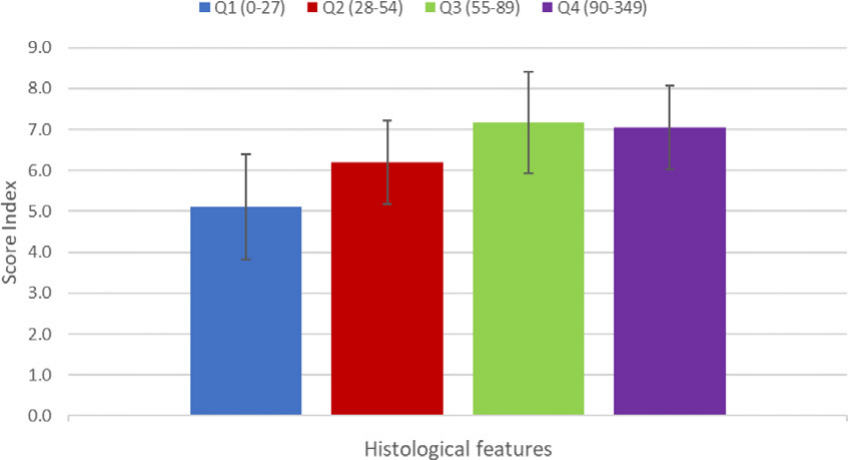
Average biopsy scores in children with different burdens of acute respiratory infection (ARI).

Histology sub-scores of 63 children whose duodenal biopsy specimens were collected, shown in Fig. 3, Fig. 4, as per diarrhoea and ARI quartiles established in the larger cohort. The histological scores were higher with increasing diarrhoeal prevalence but did not reach statistical significance (p = 0.810). The histological scores were higher in ARI Q3 and Q4 but did not reach statistical significance (p = 0.091).

## Discussion

The present study assessed the correlation of diarrhoeal and ARI morbidity with environmental enteric dysfunction and growth of children <2 years old enrolled in an existing trial cohort from Matiari, Pakistan. To our knowledge, ours is the first study globally to draw associations between diarrhoeal morbidity and duodenal histology scores indicative of EED. Our histology index was specific for duodenal biopsies, and also utilised in other cohorts.^11^ Histological changes in the gut have been associated with both diarrhoea and malabsorption, specific to regional contributors.^11^ Interestingly, while our findings did not reach statistical significance perhaps due to the relatively small sample size, the duodenal histology scores across the entire cohort were higher with ascending diarrhoeal morbidity frequency. This lends credit to empirical literature that the diarrhoeal burden among stunted children is associated with intestinal damage due to underlying EED.^14^^,^^15^ Overall, our study supports the idea that reducing the morbidity burden in infants may improve these children's overall growth parameters and EED across LMICs.

We observed that children with the highest relative frequency of diarrhoea and ARI had worse linear and ponderal growth faltering. While we did not expect diarrhoea and ARI to be singularly responsible for growth faltering in our cohort, our finding of children with the highest morbidity frequency is interesting. Growth is a complex phenomenon affected by multiple determinants including dietary factors, morbidity patterns, and environmental contributors.^1^^,^^16^^,^^17^ When the morbidity burden is particularly high as shown in Q4 of our infants, it seems to have a threshold impact on growth and drags the entire phenotypical presentation down. In children with a lower relative frequency of diarrhoea and ARI, the morbidity likely plays a role in their growth pattern but its effects are not pronounced enough to singularly explain growth faltering in these children.

There are a few limitations to this study. First, the growth parameters may have been affected due to the provision of RUSF among children with a WHZ score < −2 who failed to respond to the educational intervention at 9 months. While we did not assess any change in episodes of diarrhoea or ARI after the nutritional intervention, a similar number of children across all four quartiles of diarrhoeal and ARI morbidity received the nutritional intervention (data not shown). We further assessed different sub-groups (data not shown) and found no statistically significant difference between the nutritional intervention responders and non-responders. Second, EED scoring index histology criteria used in the study to score the duodenal biopsies is based on the EEDBI consortium, which itself is currently evolving and being simplified. However, the criteria we used to capture the degree of EED in a standardised manner in line with our current understanding of the condition.^11^ Third, we had to keep the definition of ARI days pragmatic for mothers in community-based settings in rural areas of Pakistan. We want to specify “maternal” recall bias that may occur with data collection which was mitigated to some extent by weekly reporting of the children.

Nevertheless, our study has unique strengths, including the relatively homogenous and age-matched subjects. We also focused on eliminating anthropometric measurement bias by taking readings twice which ensured accuracy and served as a quality control measure. A third reading was taken if the difference in weight was more than 50 g, length was more than 7 mm, MUAC was more than 2 mm, and occipitofrontal head circumference was more than 5 mm. Hence, the accuracy of the anthropometric data collected is a major strength of our study.

Consistently lower growth parameters of the children in our cohort were observed with the highest relative morbidity frequency. Overall, we did not find a clear correlation between diarrhoea and ARI frequency, however, the signal that the highest frequency of diarrhoea and ARI morbidity correlated with growth faltering and EED. the possibility of higher frequency of diarrheal/ARI morbidities correlating with EED can be validated in the future studies.

## Contributors

SAA conceptualised and designed the study, drafted the initial manuscript, and reviewed and revised the manuscript. AS and ZJ drafted the initial manuscript, reviewed it, and revised the manuscript. SA, FU, SJ, and AKQ coordinated, and designed the data collection instruments, and collected data. SM, NR, AR, JZM, and IM carried out the statistical analyses. NTI, SS, JI, KS, and SRM critically reviewed the manuscript for important intellectual content. All authors approved the final manuscript as submitted and agree to be accountable for all aspects of the work.

## Data sharing statement

De-identified data collected for this study are available from the corresponding author on reasonable request.

## Declaration of interests

This work was supported by the 10.13039/100000865Bill and Melinda Gates Foundation (grant number OPP1138727 to SAA and grant number OPP1144149 to SRM and The National Institutes of Health (grant number 2D43TW007585-2 to AA and SRM).
