# Supplementary material for: Impact of diarrhoea and acute respiratory infection on environmental enteric dysfunction and growth of malnourished children in Pakistan: a longitudinal cohort study
Source: Lancet Reg Health Southeast Asia. 2023 Jun 7;15:100212. doi: 10.1016/j.lansea.2023.100212 (PMC10442970; doi:10.1016/j.lansea.2023.100212)
Supplement: Supplementary Tables S1 and S2 [file mmc1.docx]

**Supplementary Table 1.** Associations at specific time points (age 0, 6, 12, 18, and 24 months) by diarrhoea and acute respiratory infection (ARI) quartiles.

|  | **Diarrhoea days quartiles** | | | | | |  |  |
| --- | --- | --- | --- | --- | --- | --- | --- | --- |
|  | **Q1 (0-27)** | **Q2 (28-54)** | | **Q3 (55-89)** | | **Q4 (90-349)** | P-values | Adjusted P-values |
| **0 month** |  | | | | |  |  |  |
| HAZ | -1.70 ± 1.26 | -1.73 ± 1.31 | | -1.62 ± 1.33 | | -1.74 ± 1.21 | 0.900 | - |
| WHZ | -1.44 ± 0.99 | -1.20 ± 1.01 | | -1.06 ± 1.00 | | -1.43 ± 1.10 | 0.045 | 0.225 |
| WAZ | -2.02 ± 1.17 | -2.00 ± 1.27 | | -1.86 ± 1.29 | | -2.14 ± 1.25 | 0.480 | - |
| **6 months** |  | | | | |  |  |  |
| HAZ | -2.24 ± 1.52 | -2.17 ± 1.43 | | -1.91 ± 1.35 | | -2.30 ± 1.44 | 0.260 | - |
| WHZ | -2.00 ± 1.27 | -2.05 ± 1.50 | | -1.76 ± 1.55 | | -2.12 ± 1.16 | 0.290 | - |
| WAZ | -2.78 ± 1.54 | -2.95 ± 1.81 | | -2.49 ± 1.62 | | -3.03 ± 1.47 | 0.120 | 0.600 |
| **12 months** |  | | | | |  |  |  |
| HAZ | -2.24 ± 1.49 | -2.35 ± 1.40 | | -2.26 ± 1.20 | | -2.46 ± 1.29 | 0.700 | - |
| WHZ | -1.71 ± 1.25 | -1.64 ± 1.20 | | -1.39 ± 1.28 | | -1.73 ± 1.14 | 0.230 | - |
| WAZ | -2.44 ± 1.48 | -2.46 ± 1.38 | | -2.15 ± 1.25 | | -2.59 ± 1.34 | 0.170 | 0.850 |
| **18 months** |  | | | | |  |  |  |
| HAZ | -2.42 ± 1.30 | -2.56 ± 1.35 | | -2.47 ± 1.08 | | -2.65 ± 1.28 | 0.660 | - |
| WHZ | -1.61 ± 1.14 | -1.65 ± 1.13 | | -1.36 ± 1.16 | | -1.70 ± 1.05 | 0.190 | 0.950 |
| WAZ | -2.30 ± 1.24 | -2.48 ± 1.29 | | -2.20 ± 1.19 | | -2.53 ± 1.19 | 0.240 | - |
| **24 months** |  | | | | |  |  |  |
| HAZ | -2.28 ± 1.32 | | -2.42 ± 1.23 | | -2.30 ± 1.13 | -2.46 ± 1.28 | 0.730 | - |
| WHZ | -1.36 ± 1.05 | | -1.31 ± 1.15 | | -1.18 ± 1.06 | -1.43 ± 0.90 | 0.410 | - |
| WAZ | -2.27 ± 1.29 | | -2.31 ± 1.31 | | -2.13 ± 1.13 | -2.44 ± 1.11 | 0.360 | - |
| **ARI days quartiles** | | | | | | | |  |
|  | **Q1 (0-14)** | **Q2 (15-48)** | | **Q3 (49-102)** | | **Q4 (103-452)** |  |  |
| **0 months** |  | | | | |  |  |  |
| HAZ | -1.49 ± 1.31 | -1.63 ± 1.20 | | -1.74 ± 1.28 | | -1.96 ± 1.28 | 0.064 | 0.320 |
| WHZ | -1.19 ± 1.00 | -1.25 ± 0.94 | | -1.30 ± 1.17 | | -1.43 ± 1.03 | 0.510 | - |
| WAZ | -1.83 ± 1.17 | -1.85 ± 1.23 | | -2.01 ± 1.15 | | -2.36 ± 1.37 | 0.008 | 0.040 |
| **6 months** |  | | | | |  |  |  |
| HAZ | -2.04 ± 1.47 | -1.99 ± 1.38 | | -2.07 ± 1.39 | | -2.50 ± 1.46 | 0.062 | 0.310 |
| WHZ | -1.94 ± 1.28 | -1.88 ± 1.51 | | -1.95 ± 1.43 | | -2.14 ± 1.27 | 0.620 | - |
| WAZ | -2.76 ± 1.60 | -2.66 ± 1.72 | | -2.75 ± 1.62 | | -3.06 ± 1.53 | 0.410 | - |
| **12 months** |  | | | | | | |  |
| HAZ | -2.38 ± 1.39 | -2.17 ± 1.28 | | -2.11 ± 1.33 | | -2.67 ± 1.28 | 0.020 | 0.100 |
| WHZ | -1.79 ± 1.05 | -1.55 ± 1.33 | | -1.48 ± 1.31 | | -1.67 ± 1.12 | 0.390 | - |
| WAZ | -2.60 ± 1.27 | -2.30 ± 1.43 | | -2.17 ± 1.41 | | -2.62 ± 1.27 | 0.082 | 0.410 |
| **18 months** |  | | | | |  |  |  |
| HAZ | -2.52 ± 1.27 | -2.54 ± 1.20 | | -2.32 ± 1.26 | | -2.77 ± 1.24 | 0.110 | 0.550 |
| WHZ | -1.69 ± 0.94 | -1.51 ± 1.28 | | -1.45 ± 1.14 | | -1.69 ± 1.05 | 0.380 | - |
| WAZ | -2.44 ± 1.06 | -2.27 ± 1.25 | | -2.22 ± 1.29 | | -2.60 ± 1.23 | 0.150 | 0.750 |
| **24 months** |  | | | | | | |  |
| HAZ | -2.40 ± 1.20 | -2.23 ± 1.18 | | -2.23 ± 1.34 | | -2.63 ± 1.17 | 0.091 | 0.455 |
| WHZ | -1.39 ± 0.93 | -1.27 ± 1.08 | | -1.24 ± 1.08 | | -1.38 ± 1.04 | 0.720 | - |
| WAZ | -2.37 ± 1.12 | -2.19 ± 1.27 | | -2.15 ± 1.23 | | -2.45 ± 1.16 | 0.280 | - |

**Supplementary Table 2.** Association of change in z-scores over time by diarrhoea and acute respiratory infection (ARI) quartiles.

| **Diarrhoea days quartiles** | | | | | |  |
| --- | --- | --- | --- | --- | --- | --- |
|  | Q1 (0-27) | Q2 (28-54) | Q3 (55-89) | Q4 (90-349) | P-values | Adjusted P-value |
| Male (n) | 67 (64.4%) | 56 (57.7%) | 61 (61.0%) | 57 (57.0%) | 0.690 | - |
| Female (n) | 37 (35.6%) | 41 (42.3%) | 39 (39.0%) | 43 (43.0%) |  |  |
| **Enrolment to 6 months** |  | | | | |  |
| ∆HAZ | -0.50 ± 1.08 | -0.46 ± 1.11 | -0.31 ± 0.96 | -0.52 ± 1.20 | 0.530 | - |
| ∆WHZ | -0.26 ± 1.49 | -0.65 ± 1.60 | -0.54 ± 1.63 | -0.68 ± 1.44 | 0.350 | - |
| ∆WAZ | -0.81 ± 1.45 | -0.95 ± 1.42 | -0.69 ± 1.38 | -0.91 ± 1.32 | 0.610 | - |
| 6 months to 12 months |  | | | | |  |
| ∆HAZ | -0.13 ± 0.58 | -0.17 ± 0.72 | -0.33 ± 0.70 | -0.13 ± 0.71 | 0.180 | 0.720 |
| ∆WHZ | 0.23 ± 1.04 | 0.30 ± 1.07 | 0.31 ± 1.15 | 0.41 ± 1.03 | 0.780 | - |
| ∆WAZ | 0.32 ± 0.78 | 0.45 ± 1.08 | 0.31 ± 0.99 | 0.57 ± 0.97 | 0.290 | - |
| 12 months to 18 months |  | | | | |  |
| ∆HAZ | -0.26 ± 0.55 | -0.17 ± 0.47 | -0.19 ± 0.43 | -0.17 ± 0.42 | 0.660 | - |
| ∆WHZ | 0.03 ± 0.80 | 0.02 ± 0.68 | 0.04 ± 0.86 | 0.05 ± 0.83 | 0.999 | - |
| ∆WAZ | 0.03 ± 0.61 | 0.03 ± 0.61 | 0.02 ± 0.68 | 0.04 ± 0.67 | 0.999 | - |
| 18 months to 24 months |  | | | | |  |
| ∆HAZ | 0.12 ± 0.35 | 0.10 ± 0.45 | 0.19 ± 0.37 | 0.23 ± 0.36 | 0.120 | 0.480 |
| ∆WHZ | 0.28 ± 0.62 | 0.31 ± 0.70 | 0.19 ± 0.66 | 0.27 ± 0.82 | 0.710 | - |
| ∆WAZ | 0.11 ± 0.44 | 0.13 ± 0.56 | 0.08 ± 0.47 | 0.15 ± 0.55 | 0.810 | - |
| **ARI days quartiles** | | | | | |  |
|  | Q1 (0-14) | Q2 (15-48) | Q3 (49-102) | Q4 (103-452) |  |  |
| Male (n) | 56 (53.8%) | 65 (63.7%) | 57 (58.8%) | 63 (64.3%) | 0.380 | - |
| Female (n) | 48 (46.2%) | 37 (36.3%) | 40 (41.2%) | 35 (35.7%) |  |  |
| **Enrolment to 6 months** |  | | | | |  |
| ∆HAZ | -0.59 ± 1.13 | -0.37 ± 1.05 | -0.34 ± 1.00 | -0.50 ± 1.17 | 0.410 | - |
| ∆WHZ | -0.66 ± 1.70 | -0.55 ± 1.45 | -0.44 ± 1.56 | -0.51 ± 1.50 | 0.870 | - |
| ∆WAZ | -0.96 ± 1.38 | -0.83 ± 1.49 | -0.74 ± 1.22 | -0.82 ± 1.47 | 0.780 | - |
| **6 months to 12 months** |  | | | | |  |
| ∆HAZ | -0.25 ± 0.57 | -0.32 ± 0.63 | -0.18 ± 0.73 | -0.05 ± 0.76 | 0.064 | 0.256 |
| ∆WHZ | 0.19 ± 1.03 | 0.27 ± 1.16 | 0.39 ± 1.04 | 0.40 ± 1.06 | 0.600 | - |
| ∆WAZ | 0.32 ± 0.86 | 0.28 ± 1.06 | 0.48 ± 0.99 | 0.59 ± 0.94 | 0.190 | 0.760 |
| **12 months to 18 months** |  | | | | |  |
| ∆HAZ | -0.18 ± 0.46 | -0.26 ± 0.46 | -0.19 ± 0.48 | -0.13 ± 0.44 | 0.380 | - |
| ∆WHZ | 0.08 ± 0.76 | 0.12 ± 0.76 | 0.02 ± 0.80 | -0.06 ± 0.84 | 0.520 | - |
| ∆WAZ | 0.11 ± 0.58 | 0.06 ± 0.63 | -0.01 ± 0.65 | -0.00 ± 0.70 | 0.650 | - |
| **18 months to 24 months** |  | | | | |  |
| ∆HAZ | 0.10 ± 0.40 | 0.23 ± 0.37 | 0.15 ± 0.36 | 0.18 ± 0.42 | 0.200 | 0.800 |
| ∆WHZ | 0.28 ± 0.58 | 0.21 ± 0.70 | 0.19 ± 0.74 | 0.35 ± 0.78 | 0.410 | - |
| ∆WAZ | 0.09 ± 0.46 | 0.08 ± 0.42 | 0.08 ± 0.56 | 0.21 ± 0.56 | 0.260 | - |
